# Relevant Factors in Adolescent Well-Being: Family and Parental Relationships

**DOI:** 10.3390/ijerph18147666

**Published:** 2021-07-19

**Authors:** Raquel M. Guevara, José E. Moral-García, José D. Urchaga, Sergio López-García

**Affiliations:** 1 Faculty of Education, Pontifical University of Salamanca, 37007 Salamanca, Spain; slopezga@upsa.es; 2 Faculty of Education Sciences, University of Seville, 41013 Sevilla, Spain; 3 Faculty of Communication, Pontifical University of Salamanca, 37007 Salamanca, Spain; jdurchagali@upsa.es

**Keywords:** adolescence, health, HRQoL, well-being, family

## Abstract

Health-related quality of life, teachers’ opinion of academic performance and self-perceived health are indicators of well-being in the adolescent stage. Some variables, such as those related to the quality of family and parental relationships, may influence these indicators and thus condition well-being during this stage of life and beyond. In this research, the aforementioned variables are analyzed jointly. It is a cross-sectional study in which 1375 adolescent schoolchildren between 11 and 18 years of age participated. Different instruments such as KIDSCREEN-10 and the questionnaire used in the international study Health Behaviour in School-Aged Children were used. The results obtained allow us to conclude that HRQoL, the teacher’s opinion of performance and the perception of health status improve as adolescents perceive a more favorable family climate, also helped by good relations between parents. Finally, it is proposed to continue with the efforts made in the school, family environment and other areas because of the enormous potential for generating quality of life in the adolescent stage and the consequent positive repercussions this has on adulthood.

## 1. Introduction

Adolescence is a key stage in the development of individuals [1]. It is a time of many physical, psychological and social changes to which children must adapt in order to reach the maturity of adulthood [2,3]. The well-being of adolescents depends on multiple factors that can determine their health-related quality of life [4], their academic performance [5] and their perception of their health status [6].

National data [7] show that more than one-third of adolescents (37.4%) consider their quality of life or well-being to be high. Boys score higher in the perception of high quality of life or well-being (43.3%) compared to girls (31.7%). This rating decreases with age in both sexes, so that while 60.5% rate their well-being as high at 11–12 years of age, the percentage drops to 19.5% at 17–18 years of age. This decline in HRQoL with age is also noted in other studies such as that of Guedes et al. with Latin American adolescents [8].

The factors that determine HRQoL in adolescents have been studied in numerous studies in recent years, most of them finding significant relationships with lifestyle habits: for example, quality of life in adolescence has been associated with physical activity and sedentary lifestyle [1,4,9,10,11] or eating habits [12]. Wu et al., for example, conducted a systematic review of cross-sectional and longitudinal studies on the subject and found evidence that a higher level of physical activity and less time spent in sedentary behavior are associated with higher HRQoL among the general population of children and adolescents [13]. Other studies point to the following as negative indicators of HRQoL in adolescence: lack of sleep, excessive workload, drug use [14], high levels of screen time [15], use of social networks [16], excess weight [17] and use of psychoactive substances [18]. However, in addition to habits or lifestyles, there are social and psychological factors that contribute to young people’s well-being and therefore influence their HRQoL. Family relationships, for example, have been shown to be an important conditioning factor of adolescent well-being [19,20,21,22], relationships between parents [23], relationships with peers [19,24] or satisfaction with school [25]. For example, the study by Sánchez-Alcaraz showed significant correlations between all dimensions of HRQoL, with students with better self-esteem and better relationships with family, friends and school having a higher quality of life [26].

In addition to HRQoL, other research points to perceived academic performance [5,27] and the perception of one’s own state of health as valuable indicators of adolescent well-being, the latter being a well-established factor in the literature [28,29] and also associated at this age with health outcomes in later stages of life [30].

Based on this background, this study sets out to analyze how the well-being of the adolescent (measured with the variables HRQL, performance and health status) is related to the different social variables (family relationships and parental relationships). Consequently, the proposed objectives are (a) to know the level of HRQL of the students, the perception that they believe their teacher has about their academic performance, the perception of their state of health and their satisfaction with the family relationships they maintain at home and relationships between their parents; (b) to study the relationship between social factors (family and parental relationships) and the selected indicators of well-being (HRQL, performance and health status); and (c) to determine which of the factors has a greater relationship with HRQoL, perceived performance and perception of their state of health.

### 1.1. Design and Participants

A descriptive cross-sectional study was designed using the survey technique. A total of 1375 adolescents took part, with a mean age of 14.44 (±1.44) years, with 50.5% being girls (*n* = 694). All of them belonged to 16 schools in Salamanca (Castilla y León, Spain) and attended between the 1st and 4th years of Secondary School in 2018. For the selection of the sample, we opted for a multistage random sampling, stratified by clusters, taking into account gender, school year and type of school (public, private). The HBSC questionnaire was distributed collectively during school time and in their usual classrooms.

### 1.2. Instruments

A sociodemographic questionnaire was used in order to determine the sex (male and female) and age in years and education level.

The following indicators were used as dependent variables:

(a) Health-Related Quality of Life Questionnaire (HRQoL). It was assessed using the KIDSCREEN-10 instrument [31], which establishes a global index of emotional well-being through 10 items that include physical, psychological and social aspects. The respondent is asked to indicate whether “in the last week” he/she felt good and fit, full of energy, sad, lonely, had enough time for him/herself, was able to do the things he/she wanted to do in his/her free time, was treated fairly by his/her parents, had fun with friends, did well at school or college and was able to concentrate or pay attention. The response, for 10 items, was on a scale of 1 to 5 where 1: never, 2: seldom, 3: sometimes, 4: often and 5: always.
(b) Self-perception of the teacher’s opinion of the student’s performance. This is an item from the HBSC questionnaire [32], in which students are asked “In your opinion, how does your teacher rate your performance at school compared to your classmates?” It has a scale with four response options where 1: very good, 2: good, 3: average and 4: below average.
(c) Students’ perception of their state of health. The assessment made by Idler and Benyamini was used, in which, after a review of 27 community studies [33], they considered this self-assessment of health to be an independent predictor of mortality. Thus, the item “would you say your health status is” was used and schoolchildren were asked to respond using a scale with 4 possible options, 1: excellent, 2: good, 3: acceptable and 4: poor.

The following indicators were used as independent variables:

(a) Family relationships. This indicator was used to analyze adolescents’ satisfaction with family relationships. An adaptation of the Cantril scale [34] was used for this purpose, consisting of a single-item question: “In general, how satisfied are you with the relationships you have in your family?” The answers are graded on a response scale ranging from 0 (very bad relationships) to 10 (very good relationships). In order to facilitate the relationship of the variables and analysis of the results, five subsequent response levels were established based on the 11 possible answers. Thus, the responses were distributed as follows:
  - Very bad relationship (initial value 0).
  - Bad relationship (initial values 1 to 4).
  - Normal relationship (initial value 5).
  - Good relationship (initial values 6 to 9).
  - Excellent relationship (initial value 10).
(b) Parental relationships. The questionnaire from the HBSC study [32] was used for this purpose, with a single item in which the respondent assesses the relationship that their parents have with each other: “In general, how do you assess the relationship that your father and mother have with each other?” The response scale ranges from 0 (very bad relationship) to 10 (very good relationship). To facilitate the relationship of the variables and analysis of the results, five subsequent response levels were established based on the 11 possible answers above. Thus, the responses were distributed as follows:
  - Very bad relationship (initial value 0).
  - Bad relationship (initial values 1 to 4).
  - Normal relationship (initial value 5).
  - Good relationship (initial values 6 to 9).
  - Excellent relationship (initial value 10).

### 1.3. Procedure

Five interviewers took part in the data collection and were responsible for passing out the questionnaires to the 16 selected schools. Prior permission was obtained from the schools, as well as informed consent from parents and legal guardians. It was made clear to the students that participation was completely voluntary, with no academic or financial compensation whatsoever. All questionnaires were administered during normal class time, preferably in tutorial sessions, and the interviewers were always present to explain and supervise the procedure.

This research respected the ethical criteria established by the Declaration of Helsinki in its 2013 revision for research of this nature, being scrupulous with the law on the protection of personal data (Organic Law 15/1999) and in accordance with current Spanish legislation on research with human beings (Royal Decree 561/1993). The study protocol was approved by the Ethics Committee for Research with Human Subjects of the Pontifical University of Salamanca.

The inclusion criteria stipulated voluntary participation and legal guardianship through the informed consent of the parents or legal guardians. The exclusion criteria were as follows: not complying with any of the previously established criteria, such as the inclusion criteria, and incorrect or incomplete answers to some of the questions in the different questionnaires.

### 1.4. Data Analysis

The statistical package SPSS version 24.0 was used to analyze the results. Descriptive analyses were carried out using mean values. The ANOVA test was used for mean comparisons, and measures of association (Eta and Eta^2^) and post hoc tests (adjusted with Bonferroni) were calculated for the analysis of difference of means.

## 2. Results

**Figure 1:**
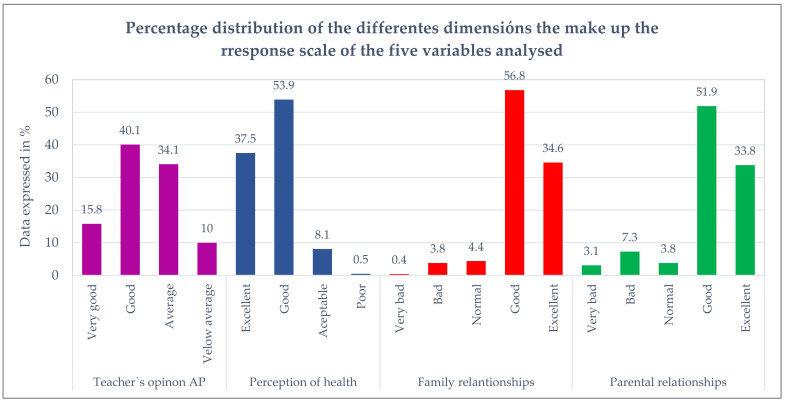
Descriptive data for the variables teacher’s opinion of AP, perception of health, family relationships and parental relationships.

Previously, a global descriptive analysis of the variables studied was performed, according to which HRQoL (measured with KIDSCREEN-10 questionnaire) showed a mean value of 37.9 (±6.08). Figure 1 shows the rest of the variables analyzed, whose descriptive results reveal that the adolescents perceive that the teacher has a good or very good opinion of their academic performance in over 55% of cases and that a large majority perceive their health positively, with 37.5% of those surveyed scoring it as excellent and 53.9% as good. Family and parental relationships are good (56.8% and 34.6%) or very good (51.9% and 33.8%).

Table 1 shows how schoolchildren who report very good family relationships have a higher HRQoL (measured with KIDSCREEN-10 questionnaire) (41.41 ± 5.05) than the rest, who rate them between good (36.63 ± 5.43) and very bad (26.00 ± 4.64), with significant differences (*p* < 0.001) in favor of those who rate their family relationships as excellent.

The students’ perception of the teacher’s opinion of their academic performance is significantly (*p* < 0.001) more favorable among those who have excellent family relationships (2.16 ± 0.84), in relation to those who have bad (2.92 ± 0.88), normal (2.78 ± 0.82) and good (2.45 ± 0.84) family relationships.

Significant differences (*p* < 0.001) were found in favor of schoolchildren who have excellent family relationships (1.46 ± 0.57), who perceive themselves to be healthier than those who state that their family relationships are bad (2.02 ± 0.77), normal (2.03 ± 0.75) or good (1.83 ± 0.58).

As a complement, we show the rest of the indicators of the ANOVA test (F), measures of association (Eta and Eta^2^) and post hoc tests (difference of means), in which we can check the specific interactions between the different variables analyzed.

Table 2 shows that the highest level of HRQoL (measured with KIDSCREEN-10 questionnaire) is significantly (*p* < *0*.001) among adolescents who perceive excellent family relationships (40.51 ± 5.50), compared to those who have good relationships (36.89 ± 5.81) or very bad relationships (34.36 ± 6.89).

It seems that parental relationships also affect the perceived opinion of teachers, with significant differences being detected (*p* < 0.001); the children of parents with excellent parental relationships (2.21 ± 0.86) consider that teachers have a better opinion of their academic performance, compared to those who consider that their parents have bad (2.74 ± 0.90) or very bad relationships (3.05 ± 0.86).

The perception of health differs significantly (*p* < 0.001) according to the relationships between parents, so adolescents who consider that their parents have excellent relationships between them have better health (1.52 ± 0.59) than children who value the relationships between their parents as normal (1.82 ± 0.62) or very bad relationships (1.90 ± 0.58).

As a complement, we show the rest of the indicators of the ANOVA test (F), measures of association (Eta and Eta^2^) and post hoc tests (difference of means), in which we can check the specific interactions between the different variables analyzed.

Finally, taking into account the previous variables and considering that this study was intended to determine which of them has the greatest weight in the prediction of HRQL, performance and health status, the results of this research show that family relationships are the best predictor (Eta^2^ = 0.051) of academic performance (according to their perception of the teacher’s opinion). Regarding the perceived health status, family relationships (Eta^2^ = 0.096) followed by parental relationships (Eta^2^ = 0.053) are the ones that correlate the most.

## 3. Discussion

Health and people’s perception of their quality of life can be influenced by multiple factors [35], such as environmental, family, school and social ones. This influence becomes especially decisive in adolescence, since there is evidence that it is a key stage in the adoption of healthy habits [28] and the strengthening of psychosocial behaviors that contribute to the improvement of adolescents’ current and future health [36].

The first aim of the research was to determine the students’ HRQoL level, perceived opinion of teachers regarding their performance and personal assessment of their health status. The results show, in line with national data [7], that more than one-third of adolescents have a high average level of HRQoL (36% of respondents score between 4 and 5 on this construct) and 91.4% perceive their health as good or excellent. The majority of adolescents (55.9%) perceive that their teacher has an excellent or good opinion of their school performance. Most of them report feeling satisfied with their family relationships as in the national study [7]. In addition, 85.7% say that relations between their parents are good or excellent.

The next objective was to analyze which of the variables is most related to HRQoL, perceived school performance and health status. Taking into account each of the variables studied, the following points can be addressed:

Family relationships are related in a relevant way in the three variables studied; thus, greater satisfaction with the relationships that occur at home is observed to favor a better HRQoL, a better perception of performance and a better state of health. In line with research, family relationships show an important influence on the health status of young people [20,21,37,38]. Parental relationships are also related in a determinant way to the well-being of the surveyed adolescents, as a positive correlation was found between parental relationships and HRQoL, performance and perception of health status. A large body of research supports this idea, confirming the relevance of positive family relationships at home during this stage as a relevant protective factor against risk behaviors and as a factor promoting psychological and social adjustment in young people [4,19,21,39]. As Alm et al. state in their 2019 research, dysfunctional family relationships can have a negative impact on children’s emotional and social competence, which in turn can hinder healthy development and ultimately affect health [39]. In addition to positive relationships with the family, in other research, positive peer relationships were found to be an important factor in adolescent well-being [40,41]; therefore, it is important that the family is also involved or ensures that adolescents have a group of equals that favors their well-being.

We can highlight that in this research family relationships are valued taking into account two types of relationships: those that occur in the home in general and the relationship that their parents have with each other. Both circumstances have shown to be of great relevance for a positive coexistence at home, which brings well-being to the adolescent at a stage in which balance, emotional support and stability are essential.

As limitations of this work, it should be noted that the HRQoL construct, despite being widely used and being a comprehensive indicator in the assessment of people’s health status, does not take into account the individual expectations of the individual in question, which may be conditioned by psychological, social, cultural, economic, demographic and other factors [42]; this may be a line of research for future work. In addition, the cross-sectional nature of this study prevents causal relationships from being established.

Another limitation of this study is that the KIDSCREEN questionnaire used includes one item related to family (was treated fairly by his/her parents) and another one related to school (did well at school or college), which have certain similarities to other variables analyzed: family relationships and student performance.

## 4. Conclusions

A higher quality of family relationships and better relationships between parents are related to a good HRQoL (measured with KIDSCREEN-10 questionnaire). Students’ perception of their teacher’s opinion of them is more positive the better their family relationships, parental relationships and subjective physical condition are. Schoolchildren consider that they are healthier the more positive their relationships with their family and between their parents are.

It is, therefore, crucial to provide students with a family environment that is as stable as possible, given the positive influence it has on their quality of life [8,19,21,43,44]. It is also known that parents have a great influence on their children and can favor the creation and consolidation of healthy habits [45,46], provide them with the necessary social and emotional stability [37,47], favor better relationships with peers [48] and consolidate habits that have transcendence in adulthood [49]. Therefore, it is essential to promote the creation of spaces where families can develop strategies that promote good communication at home. This fact will provide them with the necessary skills to create a healthy environment that helps the adolescent to develop in a positive way. Furthermore, community health promotion campaigns, from different spheres, should favor positive parenting and understand the adolescent’s social environment as a fundamental mediator, thus strengthening the synergy of actions aimed at education for health.

On the other hand, it is essential to understand the importance of the school environment in the lives of adolescents [5]. The school is an unavoidable setting, and the teacher has a great influence on the behavior of schoolchildren, not only in terms of providing learning but also as a vital example [50]. It is necessary to create a school environment that favors the stable development of students and encourages good relationships among peers [51], since it is at the school stage that a group of friends begins to be created, which favors self-esteem [52], a determining factor in the well-being and therefore the quality of life of the young person [53,54].

## Figures and Tables

**Table 1:** Descriptive data and ANOVA analysis of HRQoL, teacher’s opinion of school performance and perception of health status according to family relationships.

Quality of Family Relationships			HRQoL	Teacher’s Opinion of AP	Perception of Health
Adjusted Response Scale	Very bad relationships ^a^	M	26.00	2.83	2.00
		SD	4.64	1.16	0.89
	Bad relationships ^b^	M	32.17	2.92	2.02
		SD	6.33	0.88	0.77
	Normal relationships ^c^	M	32.46	2.78	2.03
		SD	5.99	0.82	0.75
	Good relationships ^d^	M	36.63	2.45	1.83
		SD	5.43	0.84	0.58
	Excellent relationships ^e^	M	41.41	2.16	1.46
		SD	5.05	0.84	0.57
	ANOVA	F	99.575	18.20	36.169
		Sig	<0.001	<0.001	<0.001
	Measures of association	Eta	0.475	0.225	0.309
		Eta^2^	0.225	0.051	0.096
	Post hoc Bonferroni tests (difference of means)		e > (a,b,c,d) *** d > (a,b,c) *** c > (a) * b > (a) *	e < (b,c,d) *** d < (b) ** d < (c) *	e < (b,c,d) *** d < (b) ** d < (c) *

HRQoL: health-related quality of life (measured with KIDSCREEN-10 questionnaire); M: mean; SD: standard deviation; *: *p* < 0.05; **: *p* < 0.01; ***: *p* < 0.001.

**Table 2:** Descriptive data and ANOVA analysis of HRQoL, teacher’s opinion and health perception according to parental relationships.

Quality of Parental Relationships			HRQoL	Teacher’s Opinion AP	Perception of Health
Adjusted Response Scale	Very bad relationships ^a^	M	34.36	3.05	1.90
		SD	6.89	0.86	0.58
	Bad relationships ^b^	M	35.58	2.74	1.96
		SD	6.36	0.90	0.74
	Normal relationships ^c^	M	35.90	2.44	1.82
		SD	5.93	0.92	0.62
	Good relationships ^d^	M	36.89	2.40	1.79
		SD	5.81	0.82	0.60
	Excellent relationships ^e^	M	40.51	2.21	1.52
		SD	5.50	0.86	0.59
	ANOVA	F	37.431	15.655	18.730
		Sig	<0.001	<0.001	<0.001
	Measures of association	Eta	0.318	0.212	0.231
		Eta^2^	0.101	0.045	0.053
	Post hoc Bonferroni tests (difference of means)		e > (a,b,c,d) ***	e < (d) ** e < (a,b) *** d < (a,b) *** c < (a) **	e < (a,c) ** e < (b,d) ***

HRQoL: health-related quality of life (measured with KIDSCREEN-10 questionnaire); M: mean; SD: standard deviation: **: *p* < 0.01; ***: *p* < 0.001.

## Data Availability

Data sharing is not applicable to this article.
